# Profiling and quantification of grain anthocyanins in purple pericarp × blue aleurone wheat crosses by high-performance thin-layer chromatography and densitometry

**DOI:** 10.1186/s13007-018-0296-5

**Published:** 2018-03-31

**Authors:** Stefan Böhmdorfer, Josua Timotheus Oberlerchner, Christina Fuchs, Thomas Rosenau, Heinrich Grausgruber

**Affiliations:** 1 Department of Chemistry, University of Natural Resources and Life Sciences, Vienna, Konrad Lorenz Str. 24, 3430 Tulln an der Donau, Austria; 2 Department of Crop Sciences, University of Natural Resources and Life Sciences, Vienna, Konrad Lorenz Str. 24, 3430 Tulln an der Donau, Austria

**Keywords:** Antioxidant, Chemometrics, Cluster analysis, Functional food, HPTLC, Phytonutrient, Phytochemical, Principal component analysis, Scanning densitometry, *Triticum aestivum*

## Abstract

**Background:**

Anthocyanins are abundant secondary metabolites responsible for most blue to blue-black, and red to purple colors of various plant organs. In wheat grains, anthocyanins are accumulated in the pericarp and/or aleurone layer. Anthocyanin pigmented wheat grains can be processed into functional foods with potential health benefits due to the antioxidant properties of the anthocyanins. The grain anthocyanin content can be increased by pyramidizing the different genes responsible for the accumulation of anthocyanins in the different grain layers. Our objective was to develop a high-performance thin-layer chromatography (HPTLC) method that allows the determination of both the anthocyanin profile and the total pigment concentration. Thereby, selection of breeding lines with significantly higher grain anthocyanin content from purple pericarp × blue aleurone wheat crosses should become more efficient than selection based on only visual scoring of grain color and the unspecific determination of anthocyanin concentration by UV/Vis spectroscopy.

**Results:**

A wide variability in the grain anthocyanin content was observed in breeding lines and check varieties. The highest concentration of anthocyanins was observed in deep purple (i.e. combination of the purple pericarp and blue aleurone genetics) grained breeding lines, followed by blue aleurone and purple pericarp genotypes. Determination of the total anthocyanin content was included into the chromatographic analysis, rendering an additional photometric analysis unnecessary. Ten target zones were identified in anthocyanin pigmented wheat grains; four of these zones were typically for blue aleurone types, five for purple pericarp types, and one (i.e. kuromanin glucoside) was characteristic for both. Chemometrics applied to the anthocyanin profile recorded by scanning densitometry revealed that peak heights and peak areas are highly correlated and that seven out of the ten target zones were responsible for about 90% of the total variation in the germplasm. Multivariate analysis of these seven target zones allowed not only a separation of the genetic material into purple, blue and deep purple grained genotypes, but also the identification of genotypes with a specific anthocyanin pattern. Thereby, the original classification by visual scoring was overruled in about one-third of the breeding lines.

**Conclusions:**

The presented HPTLC method with à côté calibration allowed the profiling of the pigments and quantification of wheat grain anthocyanin content in a single analysis, replacing UV/Vis spectroscopy with subsequent HPLC analysis. Moreover, no sample preparation apart from extraction and filtration is required, and more than 15 samples can be evaluated in one analysis run, corresponding to several dozens of samples per day. Hence, the method fulfills the requirements for screening methods in early generations of a plant breeding program such as high-throughput, small sample size, high repeatability, fast determination, and reasonable costs per sample. Combined with multivariate statistical analysis, the anthocyanin pattern allowed the validation of the genetic background in the offspring of purple × blue wheat crosses and, therefore, the efficient selection of genotypes exhibiting both the cyanidin and delphinidin aglycon.

## Background

Cereal grains are worldwide the major source of available carbohydrates and daily dietary energy. Cereal products prepared from refined flour or dehulled grains are consumed most frequently. However, minerals, vitamins and phytochemicals, e.g. anthocyanins, are located mainly in the outer layers of the grain [1–3], which are removed during milling as bran. Additionally, the bran fraction contains high levels of dietary fiber. Due to health promoting effects of phytochemicals and dietary fiber, an increased consumption of whole grains is recommended [4–6].

Anthocyanins are phenolic compounds (flavonoids) responsible for most blue to blue-black, and red to purple colors of diverse plant organs [7]. The interest in anthocyanins has increased in the last decades as they represent alternatives to artificial food colorants. Research also suggests potential health benefits due to their antioxidant properties [8–10]. In wheat grains, anthocyanins can be expressed in either the pericarp (i.e. purple pericarp) or the aleurone layer (i.e. blue aleurone). Higher antioxidant properties of purple- and blue-colored wheat compared to varieties without anthocyanins (i.e. white or red) were demonstrated [11, 12]. Today, anthocyanin pigmented wheat grains are processed on a limited scale into whole-grain products with specific color and taste, as well as into anthocyanin extracts for further processing into functional foods. Considering consumers´ interest in food with added health benefits, production of colored cereal varieties can be expected to increase.

**Fig. 1:**
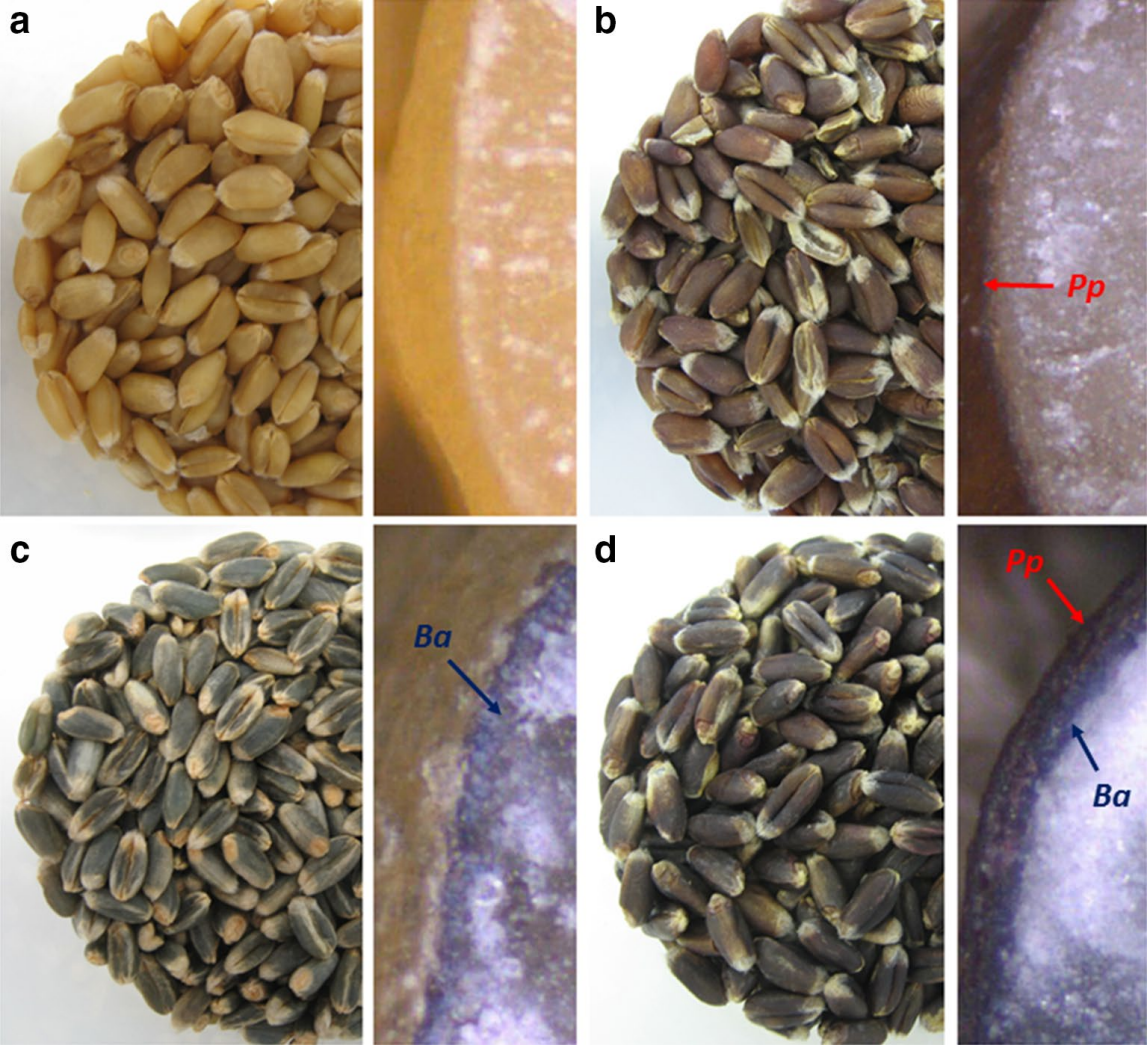
Wheat grain color due to accumulation of anthocyanins in different grain layers: **a** white (no anthocyanins), **b** purple pericarp (*Pp*), **c** blue aleurone (*Ba*), and **d** deep purple (*Ba *+ *Pp*)

To breed wheat grains with increased anthocyanin content, the genes responsible for purple pericarp (*Pp1* and *Pp3*) and blue aleurone (*Ba1* and *Ba2*) can be pyramidized (Fig. 1) by sophisticated crossbreeds. However, it is necessary to objectively evaluate the content and the composition of anthocyanins in the offspring. Currently, new hybrids are classified visually, a procedure that can be prone to subjectivity errors, while the total anthocyanin content (TAC) is determined by an unspecific photometric method. HPLC (high-performance liquid chromatography) with chemometric data analysis was used to distinguish blue aleurone, purple pericarp and ‘deep purple’ wheat genotypes according to their anthocyanin pattern [13]. The chromatographic method allowed observing variations in the content of individual anthocyanins, which is not possible by the photometric approach. HPTLC (high-performance thin layer chromatography) can replace HPLC to increase the robustness of the separation and to shorten analysis time. Applying chemometric analysis to HPTLC data, however, is challenging and requires a dedicated data preparation methodology to be successful [14, 15]. HPTLC is traditionally used for authenticity studies of medicinal plants based on the pattern of their secondary metabolites. In plant breeding, the method is hitherto not routinely used, although it was shown for chicory that it is more profitable than HPLC in the screening for sugar composition [16].

The aim of the present study was to develop an HPTLC method which allows (i) the separation of anthocyanins, (ii) the classification of samples according to their anthocyanin profile, and (iii) the determination of the total anthocyanin content of purple pericarp and blue aleurone wheat, and their hybrids.

## Methods

### Plant material

Forty winter wheat samples were tested, including 31 breeding lines, five released varieties and four genetic stocks (see Table 2 in “Appendix 1”). The germplasm was grown in 2014 under conventional farming practice in the wheat breeding nursery at the BOKU Experimental Station Groß-Enzersdorf, Lower Austria. Grain color was defined by visual scoring after harvest according to a 1 to 9 scoring scheme (see Table 2 in “Appendix 1”). According to this scheme, the samples were classified into 17 purple pericarp, 10 blue aleurone, and 13 deep purple grained genotypes.

### Sample preparation and extraction of anthocyanins

Grain samples (25 g) were milled with an AQC806 lab mill (Agromatic AG, Laupen, Switzerland). The different fractions were separated by a Promylograph LS laboratory sieving machine (Max Egger Gerätebau, St. Blasen, Austria). The bran fraction > 710 µm was collected and subsequently milled with a Cyclotec™ 1093 mill (Foss GmbH, Austrian subsidiary, Vienna) equipped with a 1 mm sieve. The milled samples were stored in a freezer at − 20 °C. The moisture content of the bran samples was measured with a MA35 moisture analyzer (Sartorius, Göttingen, Germany) and was typically 10%.

All samples were extracted according to Abdel-Aal and Hucl [17]. In brief, 8 mL of methanol and 1 M HCl (85:15, v/v) were added to 1 ± 0.002 g of milled bran in a 15 mL centrifuge tube. The tubes were shaken shortly by hand and then agitated in an overhead shaker (Heidolph Instruments, Schwabach, Germany) for 30 min at 150 rpm. The tubes were centrifuged for 5 min at 4000 rpm in a Z206A compact centrifuge (Hermle, Wehingen, Germany). The supernatant was decanted and filled to 6 mL with the extraction solvent. The extracts were stored in a freezer at − 20 °C protected from light to be analyzed within days.

### High-performance thin-layer chromatography

For all analyses, HPTLC plates (200 × 100 mm, 200 µm silica gel 60 F_254_, glass plates; Merck, Darmstadt, Germany) were used. Samples—thawed and filtrated through a 0.45 µm PTFE syringe filter (VWR International, Darmstadt, Germany) into amber vials—and anthocyanin standards, i.e. kuromanin (cyanidin 3-*O*-glucoside) chloride and myrtilin (delphinidin 3-*O*-glucoside) chloride (Extrasynthese, Genay, France), were applied with an Automatic TLC Sampler 4 (ATS 4, CAMAG, Muttenz, Switzerland), using the following settings for 18 sample tracks per plate: band length 8.0 mm, track distance 10.0 mm, dosage speed 150 nL s^−1^, first application position: 15 mm from the left edge (x-axis), 8 mm from the bottom edge (y-axis); 14 µl of each sample and standard were applied. Development was carried out in an Automated Developing Chamber (ADC2, CAMAG, Muttenz, Switzerland) with the following settings: migration distance 60 mm, 20 min saturation, 10 min activation using molecular sieve for a relative humidity of 0–5%; 5 min drying time. The same solvent mixture was used for both plate development (10 mL) and saturation (25 mL): ethyl acetate:2-butanol:1 mM trifluoroacetic acid in methanol:water (3:2:1:1, v/v).

Scanning densitometry was performed with a TLC Scanner 3 (CAMAG, Muttenz, Switzerland) both directly after sample application and after development at 535 nm using the following settings: scanning speed: 20 mm s^−1^; data resolution: 100 µm per step; slit: 5 × 0.2 mm, micro. All instruments were controlled with VisionCats 2.0 software (CAMAG, Muttenz, Switzerland).

**Fig. 2:**
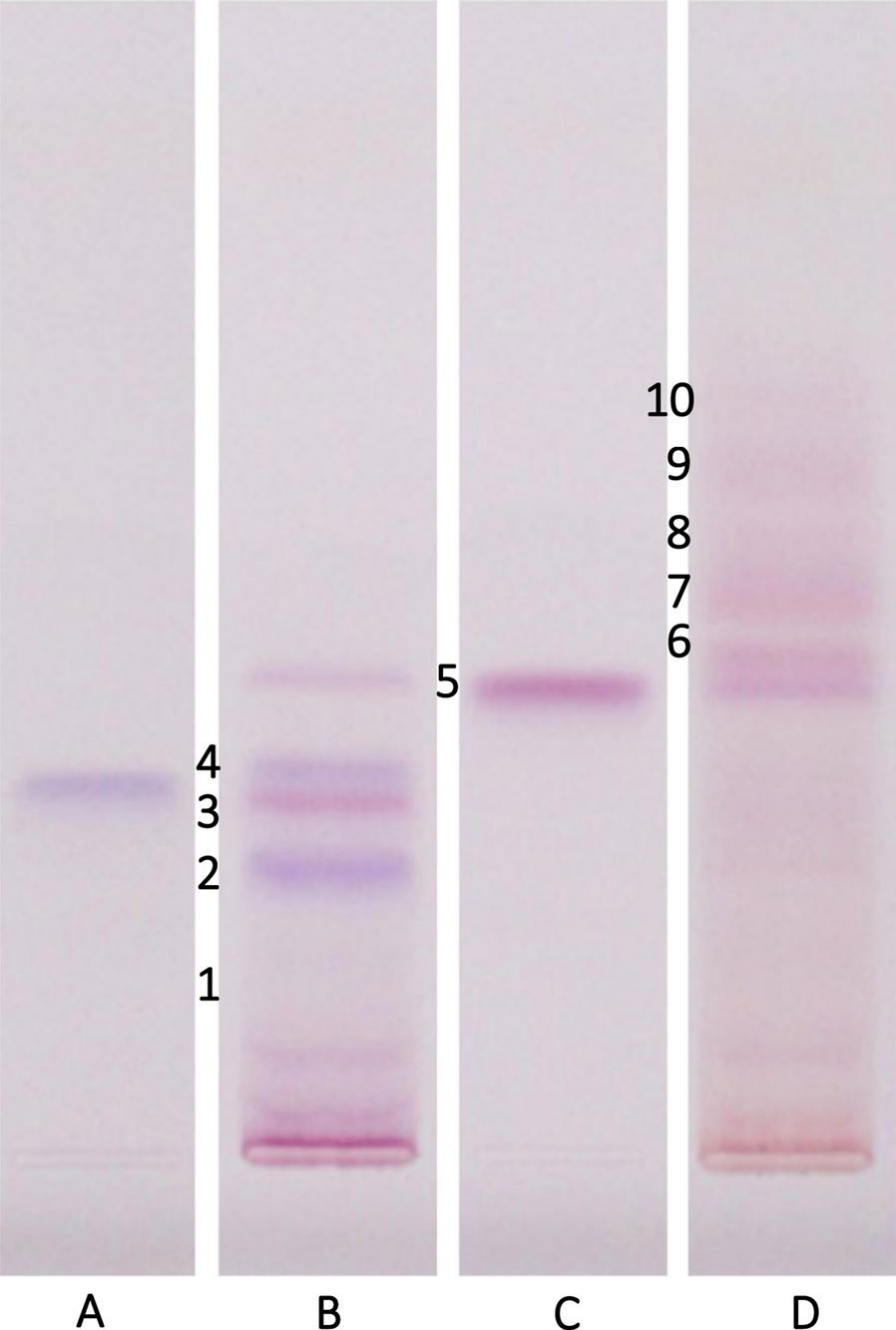
Chromatograms of wheat bran anthocyanins and anthocyanin standards: **A** myrtilin chloride; **B** blue aleurone wheat; **C** kuromanin chloride; **D** purple pericarp wheat. The ten target zones are indicated by numbers. Contrast of the image was adjusted with VisionCats software to improve clearness

Peak heights (intensities) and peak areas were evaluated with winCATS 1.4.9 software (CAMAG, Muttenz, Switzerland). Peaks with intensities less than 2 AU were ignored and only peaks with a retention factor (*R*_*f*_) between 0.2 and 0.7 were considered. In total, ten target zones at *R*_*f*_ 0.2, *R*_*f*_ 0.28, *R*_*f*_ 0.34, *R*_*f*_ 0.37, *R*_*f*_ 0.45, *R*_*f*_ 0.49, *R*_*f*_ 0.52, *R*_*f*_ 0.59, *R*_*f*_ 0.65 and *R*_*f*_ 0.70 were obtained (exemplified in Fig. 2). Shifts in *R*_*f*_ between plates were small and easily corrected by the two anthocyanin standards and four check samples which were included in each plate. Peak heights and areas were used for statistical analysis.

TAC determination with à côté calibration was conducted according to Oberlerchner et al. [18]. In brief, kuromanin chloride standards were applied to each plate outside the area required for chromatography (70 mm, y-axis), while the samples were applied at their usual position near the bottom edge (8 mm, y-axis). The plates were scanned at 535 nm directly after application of the standards and then again after sample application. Then, chromatography was performed as described above. The peak areas of the standards acquired in the first scan were used for calibration, establishing a linear relationship of the square root of the peak area and the decimal logarithm of the kuromanin concentration. This calibration was then used to convert the areas of the application spots, which had been determined in the second scan, into kuromanin-equivalents (kur-eq) per gram bran.

### Statistical analysis

Procedure MIXED of SAS 9.4 software (SAS Institute Inc., Cary, NC) was used for mixed analysis of variance with grain color as fixed effect. The Tukey–Kramer method was applied to compare the least square means to account for the differences in the number of genotypes per grain color class. Principal component analysis (PCA) was executed for dimensionality reduction via the BIPLOT macro [19]. Hierarchical cluster analysis using Euclidian distances for the similarity matrix and average linkage as algorithm for cluster formation was carried out using Genstat 18^th^ ed. Software (VSN International Ltd, Hemel Hempstead, UK). The whole workflow of the method is demonstrated in Fig. 6 (“Appendix 2”).

## Results

### Total anthocyanin content

Total anthocyanin contents (TAC) in the investigated bran samples ranged from 47.5 to 1289.6 µg g^−1^ (Table 1 and [18]). Analysis of variance revealed significant differences between grain color classes. The lowest TAC was observed for purple grained check varieties ‘Rosso’, ‘Charcoal’ and ‘Konini’, whereas the highest TAC was recorded for ‘deep purple’ colored breeding lines (see Table 2 in “Appendix 1”). Within blue and purple grained genotypes, breeding lines showed a tendency to higher TAC compared to check varieties (released varieties and genetic stocks). However, these differences were not statistically significant at *p *= 0.05 (Table 1).

**Table 1:** Variation in the total anthocyanin content (TAC, kur-eq) of bran of anthocyanin pigmented wheat breeding lines and check varieties

Source	Seed colour^1^	TAC_VS_ (µg g^−1^ db)^2^			TAC_HPTLC_ (µg g^−1^ db)^3^		
		Min	Max	Mean^4^	Min	Max	Mean^4^
Lines	Ba + Pp	359.9	1289.6	780.5 a	466.8	1289.6	880.9 a
Lines	Ba	161.5	879.2	496.2 ab	295.1	679.3	496.3 b
Checks	Ba	117.6	669.0	353.8 bc	117.6	669.0	353.8 bc
Lines	Pp	124.3	502.5	219.7 bc	124.3	437.7	221.0 c
Checks	Pp	47.5	174.7	100.3 c	47.5	174.7	100.3 c

^1^Ba, blue aleurone; Ba + Pp, deep purple (blue aleurone + purple pericarp); Pp, purple pericarp

^2^TAC in grain color classes based on visual scoring

^3^TAC in grain color classes based on chemometrics of the anthocyanin profile

^4^Means with different letters are significantly different at *p *= 0.05 (Tukey–Kramer test)

**Table 2:** Investigated wheat germplasm and the respective total anthocyanin content (TAC, kur-eq) in the bran fraction (> 710 µm particle size)

Genotype	Status^1^	ID	Seed color^2^	TAC (µg g^−1^)
Amethyst (AGG29407)	CV	AMY	4	132.2
Charcoal (CItr17422)	GS	CCL	5	74.0
Indigo (BVAL216120)	CV	IND	4	174.8
Konini (TRI16816)	CV	KON	4	79.3
Otello (J.1083)	BL	OTE	4	93.8
Rosso (SZD6758)	CV	ROS	4	47.5
276-OTE/TBK-P2512.5G.14	BL	p1	4	160.9
327-SB3/REN//CCL-P2415.6G.14	BL	p2	5	195.8
345-SB1//SAT/IND-P3001.1K.14	BL	p4	5	502.5
354-IND/TBK-P2903.1G.14	BL	p6	4.5	188.7
374-IGR/TBK//SB1/IND-P2722.1G.14	BL	p7	4	185.1
376-OTE/SB2-P2417.1K.14	BL	p8_x_	4	185.1
376-OTE/SB2-P2418.1K.14	BL	p8_y_	4	202.4
390-SB2/TBK//IND-P2704.1K.14	BL	p10	5	309.7
391-IND/KAR//SB2/TBK-P2724.1G.14	BL	p11	4	124.3
392-SB2/TBK//AMY-P2705K.14	BL	p12_x_	3.5	148.6
392-SB2/TBK//AMY-P2709.6G.14	BL	p12_y_	5	213.0
Sebesta Blue 1 (PI634538)	GS	SB1	7	380.1
Sebesta Blue 2 (PI634539)	GS	SB2	7	535.6
Skorpion (01C0106994)	CV	SKO	6	150.1
Tschermaks Blaukörniger (BVAL214025)	GS	TBK	6	399.3
276-OTE/TBK-BA2502.2G.14	BL	b1	6	295.1
327-SB3/REN//CCL-BA2820.3G.14	BL	b2	7	588.7
346-SB1/IND-BA2907.1G.14	BL	b5	7	879.2
354-IND/TBK-BA2902.5G.14	BL	b6	7	161.7
392-SB2/TBK//AMY-BA2706.5G.14	BL	b12_x_	7	373.4
392-SB2/TBK//AMY-BA2710.4.14	BL	b12_y_	7	679.3
276-OTE/TBK-DP2503.3G.14	BL	d1	9	1276.2
327-SB3/REN//CCL-DP2407G.14	BL	d2	9	592.8
339-TBK/IND-DP2606.1G.14	BL	d3_x_	8	466.8
339-TBK/IND-DP2608.6G.14	BL	d3_y_	8.5	761.2
345-SB1//SAT/IND-DP3002.6K.14	BL	d4	9	1289.6
346-SB1/IND-DP2908.6G.14	BL	d5_x_	8.5	992.7
346-SB1/IND-DP2909.1G.14	BL	d5_y_	8.5	866.4
354-IND/TBK-DP2906.6G.14	BL	d6	9	606.8
389-SB1/IGR//AMY-DP2522.1G.14	BL	d9	8	437.7
390-SB2/TBK//IND-DP2701.2K.14	BL	d10_x_	9	1177.2
390-SB2/TBK//IND-DP2702.6K.14	BL	d10_y_	9	889.0
392-SB2/TBK//AMY-DP2707.3K.14	BL	d12_x_	9	430.2
392-SB2/TBK//AMY-DP2714.1.14	BL	d12_y_	8	359.8

^1^BL, breeding line; CV, cultivated variety; GS, genetic stock

^2^Visual scores of seed colors: 1, white; 2, light red pericarp; 3, red pericarp; 4, light purple pericarp; 5, purple pericarp; 6, light blue aleurone (greyish seed); 7, dark blue aleurone; 8, heterogenous deep purple (< 50% greyish blue); 9, deep purple/black (blue aleurone and purple pericarp)

### Multivariate statistics of HPTLC data

PCA of the ten selected peaks revealed that peak heights and peak areas are highly associated: both the length of their vectors are similar and the angle between the vectors is small (see Fig. 7 in “Appendix 2”). Correlation analyses confirmed the relationship between peak height and peak area. Correlations coefficients ranged from *r* = 0.95 (*p* < 0.0001) for target zone 4 to *r* = 0.995 (*p* < 0.0001) for target zones 3 and 6. The two biplot axes explained 52.2 and 29.7% of the total variation and the grain color classes were distributed as follows: blue aleurone genotypes along vectors of target zones 1 to 4, purple pericarp types along the vectors of target zones 6 to 10, and deep purple types in between these groups. Target zone 5, which corresponds to kuromanin glucoside (see Fig. 2), showed an intermediate position to the other two groups of target zones. Removing peak areas from PCA resulted in a negligible improvement with respect to explained variation by the first two principal components (82.2%) (see Fig. 8 in “Appendix 2”). Moreover, peaks at the beginning and end of the chromatogram (target zones 1, 9 and 10 at *R*_*f*_ 0.2, 0.65 and 0.7, respectively) were either less important concerning differentiation of the germplasm—as visible by the shorter vector length—or were highly associated with other peaks (e.g. target zone 1 with 4, 9 with 7 and 10 with 8). Therefore, these three peaks were also removed from PCA.

**Fig. 3:**
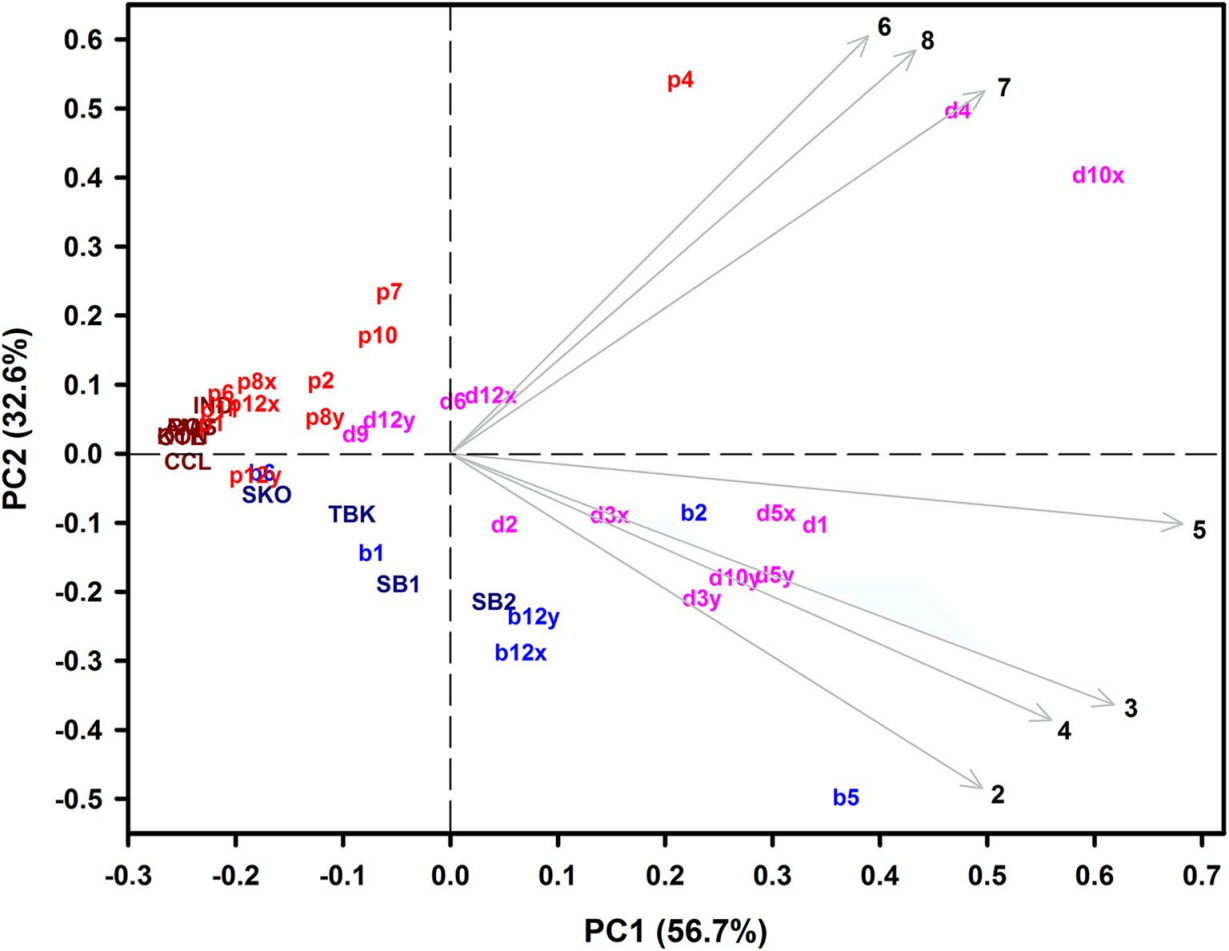
PCA biplot of seven anthocyanin HPTLC peak heights of blue, purple and deep purple grained wheat genotypes. Peak numbers 2 to 8 correspond to the target zones at the following *R*_*f*_ values: 0.28, 0.34, 0.37, 0.45, 0.49, 0.52 and 0.59, respectively. Genotype codes correspond to Table 2 in “Appendix 1”

The final PCA with the peak heights of the remaining seven peaks improved the explained variation significantly: PC1 and PC2 explained 56.7 and 32.6%, respectively (Fig. 3). A grouping of grain color classes is obvious, but variation in the breeding lines is considerable within grain color. Two breeding lines are grouped differently to visual scoring: purple grained line p12y is—apart from its sister line p12x—overlapping with blue grained genotypes and blue grained b2 is located in the group of deep purple grained genotypes.

**Fig. 4:**
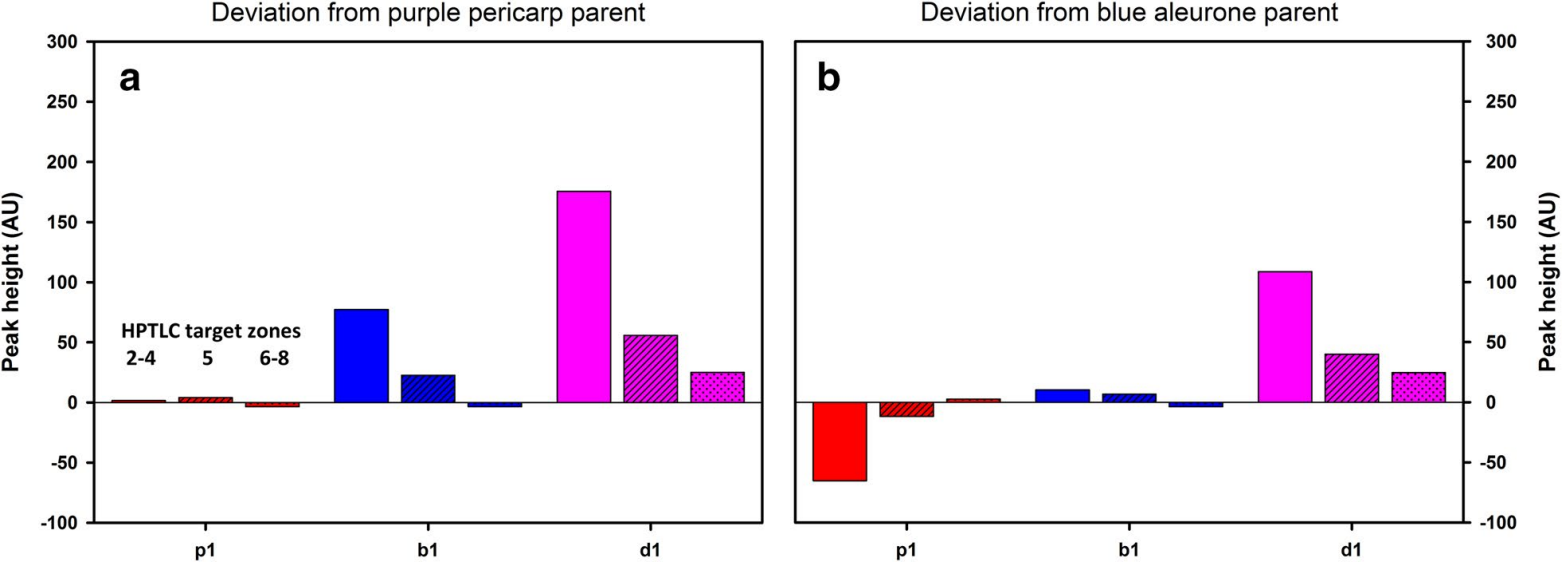
Deviations of selected breeding lines of cross 276 from their purple pericarp (**a**) and/or blue aleurone (**b**) parent in their anthocyanin pattern (sum of peak heights in chromatogram target zones 2 to 8, corresponding to *R*_*f*_ values 0.28, 0.34, 0.37, 0.45, 0.49, 0.52 and 0.59). Genotype codes correspond to Table 2 in “Appendix 1”

From both the chromatogram (Fig. 2) and the biplot (Fig. 3) it is obvious that target zones 2 to 4 and 6 to 8 are characteristic for blue aleurone and purple pericarp genotypes, respectively, while target zone 5 (kuromanin) is present in both genetic backgrounds. Investigating the breeding lines for their deviations in the anthocyanin pattern from their purple and blue parents provides additional information. For example, the purple (p1), blue (b1) and deep purple (d1) lines of ‘cross 276’ (Table 2 in “Appendix 1”) show an expected pattern: p1 and b1 are similar to their respective parents and show no deviations in the anthocyanin pattern, while d1 is a combination of both the purple and blue pattern (Fig. 4). This result is also obvious in the biplot (Fig. 3) where p1 and b2 are nearby the purple and blue check and parent varieties and d1 lies apart from them in the deep purple group.

**Fig. 5:**
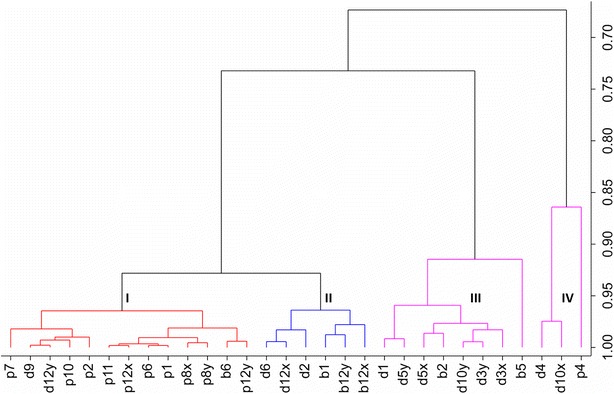
Hierarchical clustering (average linkage) of breeding lines based on the deviations from their anthocyanin colored parent with regard to peak height of key target zones 2 to 4, 5, and 6 to 8. Genotype codes correspond to Table 2 in “Appendix 1”

**Fig. 6:**
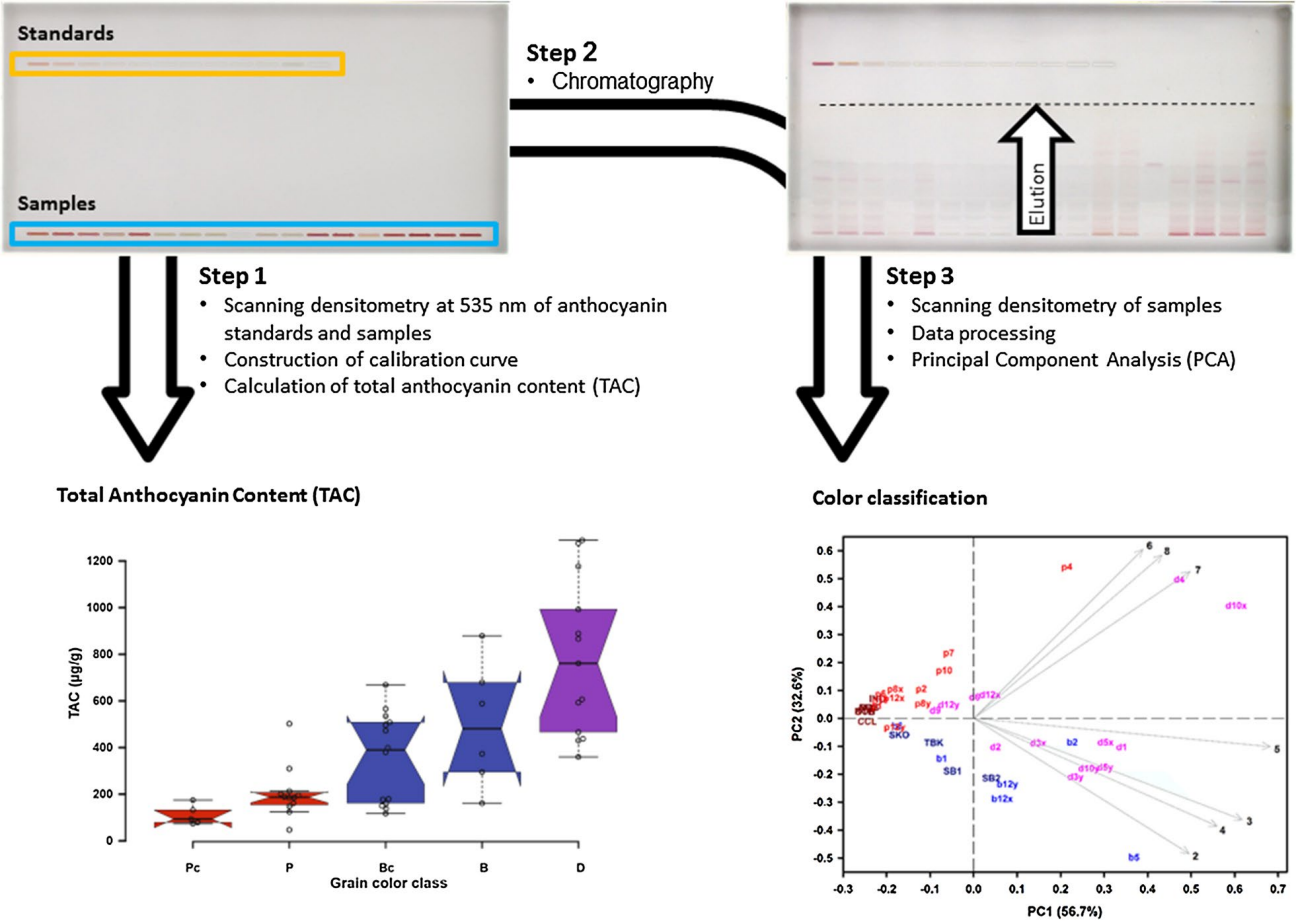
Workflow of the HPTLC analysis. In the first step, peak areas of samples and à côté standards are measured by scanning densitometry. This data is used for TAC determination. In step 2, the plates are developed and the anthocyanins separated. In step 3, the chromatograms are recorded by scanning densitometry, generating the data used for PCA

**Fig. 7:**
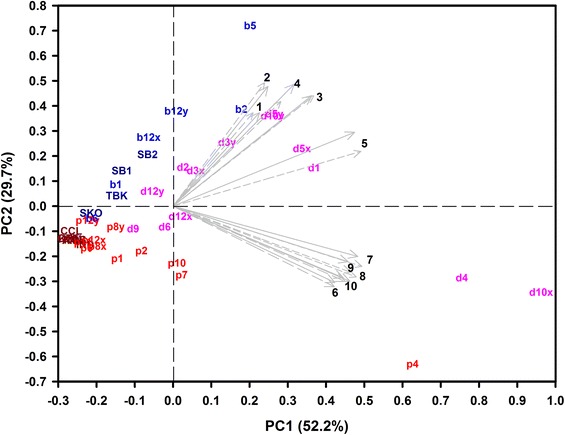
PCA biplot of HPTLC peak heights and peak areas of blue, purple and deep purple grained wheat genotypes. Vector numbers correspond to the ten target zones at *R*_*f*_ 0.2, 0.28, 0.34, 0.37, 0.45, 0.49, 0.52, 0.59, 0.65 and 0.7, respectively. Genotype codes correspond to Table 2 in “Appendix 1”. Vectors for peak heights and peak areas are indicated by solid and dashed grey lines, respectively

**Fig. 8:**
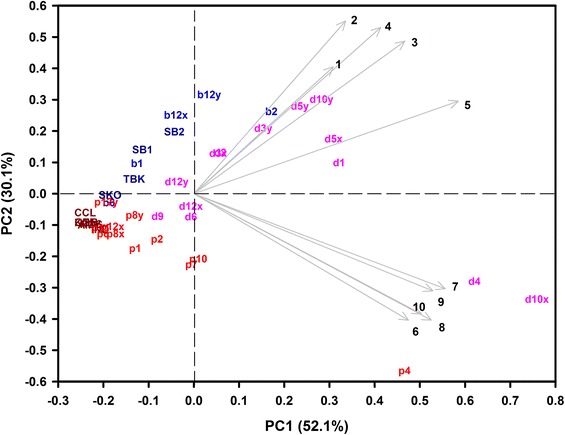
PCA biplot of HPTLC peak heights only of blue, purple and deep purple grained wheat genotypes. Vector numbers correspond to the ten target zones at *R*_*f*_ 0.2, 0.28, 0.34, 0.37, 0.45, 0.49, 0.52, 0.59, 0.65 and 0.7, respectively. Genotype codes correspond to Table 2 in “Appendix 1”

**Fig. 9:**
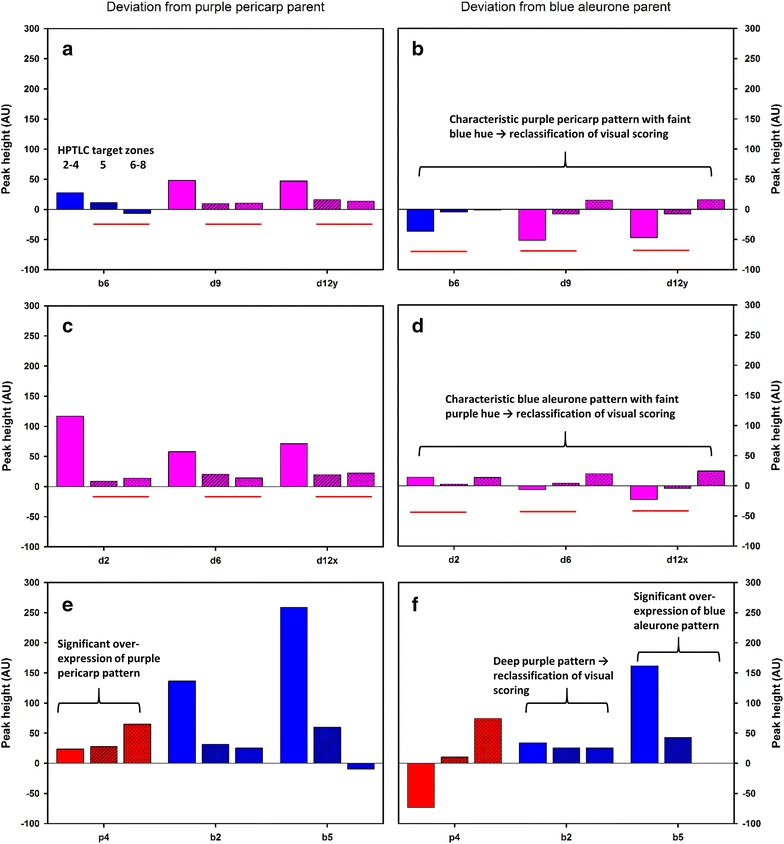
Deviations of selected breeding lines from their purple pericarp (**a**, **c**, **e**) and/or blue aleurone (**b**, **d**, **f**) parent in their anthocyanin pattern (sum of peak heights in chromatogram target zones 2 to 4, 5, and 6 to 8, corresponding to *R*_*f*_ values 0.28, 0.34, 0.37, 0.45, 0.49, 0.52 and 0.59). Subplots **a** and **b**, **c** and **d**, and **e** and **f** include genotypes included by cluster analysis in Cluster I (purple pericarp), Cluster II (blue aleurone) and Clusters III and IV (deep purple) but which were visually classified otherwise

Cluster analysis of breeding lines based on their deviation in peak heights from their colored parents in the three key target zones (i.e. 2 to 4, 5, and 6 to 8) revealed four clusters (Fig. 5). Cluster I and II include breeding lines with an anthocyanin pattern typical for purple pericarp and blue aleurone germplasm, respectively. Cluster III includes deep purple lines with a characteristic accumulated pattern of both genetic backgrounds, whereas Cluster IV includes deep purple breeding lines with an expression of anthocyanins in key target zones higher than expected from the performance of their parents. In each cluster, breeding lines are included which were visually scored differently from their grouping by their anthocyanin pattern, i.e. b6, d9 and d12y in Cluster I, d2, d6 and d12x in Cluster II, b2 and b5 in Cluster III, and p4 in Cluster IV (Fig. 6). The deviations in the anthocyanin pattern (see Fig. 9 in “Appendix 2”) confirmed the grouping by cluster and principal component analysis and allowed a reclassification of the visual scoring. In total, 30% of the breeding lines were not correctly evaluated by the visual scoring. Reclassification of the grain color based on the multivariate statistics led also to a better differentiation of the grain color classes in the analysis of variance and post hoc mean (TAC_HPTLC_) comparisons (Table 1).

## Discussion

### Grain anthocyanin concentration

The variability in TAC observed in this study is comparable to almost all previous studies. In wheat bran of a purple and blue grained wheat grown from 1996 to 1998 in Canada on average 235.9 and 452.9 µg g^−1^, respectively, were reported [20]. Similar values were stated by the same group in later studies: 321 and/or 405 µg g^−1^ for blue wheat bran [21] and 154 to 285 µg g^−1^ for purple wheat bran [22]. Siebenhandl et al. [1] observed 168.6 and 225.8 µg g^−1^ for the bran and shorts fraction of Austrian purple and blue wheat, respectively. In the bran of a commercial sample of purple wheat, 295 µg g^−1^ were determined [23]. Contrariwise, for a commercial purple wheat bran sample from Canada, a significantly higher TAC of 1155 µg g^−1^ was reported [24], a value which was reached in the present study only by a few deep purple grained lines. Differences in TAC across the literature can be explained by different germplasm, environmental conditions and methodology, e.g. different mills and mesh widths used for the fractionation of bran.

Although the purple pericarp and blue aleurone traits are inherited by only two major genes each [25, 26], several studies revealed not only significant genotypic effects for TAC, but also significant genotype by environment interactions, and significant interactions between environmental factors (years, locations, management) [22, 27, 28]. Anthocyanin accumulation in the grains of various purple and blue wheat varieties increases with grain development and significantly declines during the hard dough stage [29–31]. Therefore, harvesting germplasm at different maturity stages, which in practice often happens in segregating material, can have influence on the genotype by environment interaction. Increased TAC values were observed in earlier harvested samples of one and the same genotype, and even grain position can have an effect on grain anthocyanin concentration [27]. Such environmental influence is most probably also responsible for some false classifications of seed color by visual scoring in the present study. The environmental influence on concentrations and composition of anthocyanins in plant organs was also demonstrated for other crops, such as maize [32], potatoes [33], grape [34] and *Vaccinium* berries [35].

A higher TAC in breeding lines compared to parental check varieties was observed not only in this study but also by other researchers [13, 22, 30, 36, 37]. This can be explained by the conscious selection of crossing progenies to the respective environmental conditions, whereas the parental donors of grain color are often non-adapted or even ‘exotic’ genotypes developed elsewhere.

Appropriate milling, debranning and fractionation techniques can be used to recover mill streams with increased contents of phytonutrients [23, 38, 39] for the production of functional foods. With respect to anthocyanin pigmented wheat it has to be considered that blue aleurone and deep purple grained types contain high amounts of antioxidants in the aleurone. To this end, techniques capable of exploiting and/or separating also the aleurone layer have to be applied [40–42].

TAC was determined by HPTLC à côté calibration [18] which was shown to be highly correlated to the unspecific UV/Vis spectroscopic method. A determination of TAC after chromatography, e.g. by integrating the total peak area at 535 nm, was not possible as the anthocyanins decolorized during development. Recently, Łata et al. [43] confirmed that anthocyanins are prone to decomposition on silica plates. For the investigated samples, the total peak areas after development were well below 50% of the area of the application spots. It was therefore necessary to measure the peak areas of the application spots before chromatographic separation, offering thereby also an opportunity to increase sample throughput [18].

### Classification by anthocyanin pattern

It was previously shown that genotypes of anthocyanin pigmented wheat could be identified according to their HPLC chromatograms [13], which required about 70 min per sample or 20 samples per day, not considering sample preparation to prevent premature clogging of the column. The identification of plants by their characteristic pattern of compounds is one of the main applications of HPTLC [44]. It has advantages over the column-based HPLC considering robustness as contaminations of the stationary phase do not interfere with subsequent analyses which reduces the necessary sample preparation to filtration. Also sample throughput is higher, as several samples are developed on the same plate in parallel. Twenty samples—a day’s worth of samples of the HPLC method—can be analysed on a single plate within less than 2 h without difficulty (see Table 3 in “Appendix 1”). The effective analysis time per sample is therefore in the range of a few minutes, making HPTLC an ideal tool for screening campaigns [16]. The decision about a plant’s identity is generally supported by visual inspection of the chromatogram [45], and efforts have been made to replace this practice with the more objective evaluation of chromatograms by PCA [46].

**Table 3:** Used resources for the presented HPTLC method compared to a previously reported method combining HPLC and photometry [13]

	HPLC with photometry	HPTLC
Sample amount	1 g wholemeal flour	25 g grain, 1 g bran
Solvent used for extraction	25 mL	6 mL
Extraction time^1^	108 min	36 min
Analysis time per sample	62 min HPLC 1 min photometry	6 min for both analyses
Costs for stationary phase per sample	4–6 €^2^	0.5 €
Consumed mobile phase per sample	14.694 mL in total^3^ 12.106 mL water 1.147 mL acetonitrile 1.147 mL methanol 0.294 mL formic acid	1.944 mL in total^4^ 0.833 mL ethyl acetate 0.555 mL 2-butanol 0.287 mL 1 mM trifluoroacetic acid in methanol 0.287 mL water
Consumed sample volume	2 µL for chromatography^5^ 200–2000 µL for photometry^6^	14 µL for both analyses^5^
Time for data evaluation	Approximately the same for both protocols	

^1^Several samples can be extracted simultaneously

^2^Replacing the column after 150 to 200 injections

^3^HPLC grade

^4^Analytical grade

^5^Not accounting for losses in the sampler; a larger volume is necessary to reach the required filling level in the vial

^6^Sample used for photometry can be recovered

The result of an HPTLC analysis is usually documented by either video or scanning densitometry. In video densitometry, a picture of the plate is taken with a digital camera under illumination with white light or UV light. These pictures resemble the visual impression of the plate and contain information on both the position and the color of the spots. However, chromatograms extracted from these pictures lack both in resolution and in sensitivity [47]. Several approaches have been published to use data obtained this way for PCA, but only in a few cases satisfactory results were obtained [48]. While irregularities in chromatograms (e.g. uneven solvent fronts, irregular illumination of the plate) can be compensated [14, 49], the low sensitivity of video densitometry can barely be improved by data manipulation. Faint peaks are easily obscured by random noise and are consequently not recognized by PCA [46]. In a previous study, PCA of anthocyanins according to video densitometric data regularly revealed random factors or variations between plates as main principal components [50]. In scanning densitometry, the plate is illuminated by monochromatic light, and the reflected light is detected by a photomultiplier. This allows a sensitive and wavelength-dependent detection of analytes on the plate. In contrast to video densitometry, noise and variations between plates are greatly reduced, resulting in the reproducible detection even of faint spots. In the data obtained this way, signals can be differentiated more clearly from noise than in video densitometry [51], providing more meaningful input data for statistical analyses. This data still needs to be checked for chromatographic irregularities, such as shifting retention factors, and corrected if required. In the present study it was also shown that the removal of some key target zones from PCA improved the differentiation as the removed peaks didn’t contribute significantly to the differentiation of the material and, moreover, were highly correlated to other peaks. This strong association between some peaks might be connected to the recently recognized decomposition of anthocyanins on silica plates, which forms anthocyanidin aglycons from glycosidated anthocyanins [43].

## Conclusions

The presented HPTLC method with à côté calibration combines both reliable quantification and profiling of wheat grain anthocyanins into one analysis. Compared to other chromatographic methods, the method is highly productive and suitable for breeding programs with several dozens of samples per working day and offers a significantly better cost efficiency. Chemometric analysis of data obtained by scanning densitometry was highly efficient in confirming or questioning the grain color determined by visual scoring. For almost one-third of the breeding lines a reclassification of the visually assessed grain color was necessary after HPTLC. Moreover, a few genotypes were identified which exhibited an anthocyanin pattern not expected according to the involved colored parents. This germplasm is of special interest for further studies on the spatial and temporal biosynthesis of anthocyanins in the wheat grain and its genetic regulation.

## Appendix 1

See Tables 2 and 3.

## Appendix 2

See Figs. 6, 7, 8 and 9.

## Abbreviations

Ba: blue aleurone
db: dry base
HPLC: high performance liquid chromatography
HPTLC: high performance thin layer chromatography
kur-eq: kuromanin equivalents
PCA: principal component analysis
Pp: purple pericarp
R_f_: retention factor
TAC: total anthocyanin content

## References

[1] Siebenhandl S, Grausgruber H, Pellegrini N, Del Rio D, Fogliano V, Pernice R, Berghofer E (2007). Phytochemical profile of main antioxidants in different fractions of purple and blue wheat, and black barley. J Agric Food Chem.

[2] Surget A, Barron C (2005). Histologie du grain de blé. Ind Céréal.

[3] Onipe OO, Jideani AIO, Beswa D (2015). Composition and functionality of wheat bran and its application in some cereal food products. Int J Food Sci Technol.

[4] Aune D, Norat T, Romundstad P, Vatten LJ (2013). Whole grain and refined grain consumption and the risk of type 2 diabetes: a systematic review and dose–response meta-analysis of cohort studies. Eur J Epidemiol.

[5] Seal CJ, Brownlee IA (2015). Whole-grain foods and chronic disease: evidence from epidemiological and intervention studies. Proc Nutr Soc.

[6] McRae MP (2017). Health benefits of dietary whole grains: an umbrella review of meta-analyses. J Chiropractic Med.

[7] Schwinn KE, Davies KM, Davies KM (2004). Flavonoids. Plant pigments and their manipulation.

[8] Kähkonen MP, Heinonen M (2003). Antioxidant activity of anthocyanins and their aglycons. J Agric Food Chem.

[9] Smeriglio A, Barreca D, Bellocco E, Trombetta D (2016). Chemistry, pharmacology and health benefits of anthocyanins. Phytother Res.

[10] Li D, Wang P, Luo Y, Zhao M, Chen F (2017). Health benefits of anthocyanins and molecular mechanisms: update from recent decade. Crit Rev Food Sci Nutr.

[11] Li W, Shan F, Sun S, Corke H, Beta T (2005). Free radical scavenging properties and phenolic content of Chinese black-grained wheat. J Agric Food Chem.

[12] Hu C, Cai YZ, Li W, Corke H, Kitts DD (2007). Anthocyanin characterization and bioactivity assessment of a dark blue grained wheat (*Triticum aestivum* L. cv. Hedong Wumai) extract. Food Chem.

[13] Syed Jaafar SN, Baron J, Siebenhandl-Ehn S, Rosenau T, Böhmdorfer S, Grausgruber H (2013). Increased anthocyanin content in purple pericarp × blue aleurone wheat crosses. Plant Breed.

[14] Audoin C, Holderith S, Romari K, Thomas OP, Genta-Jouve G (2014). Development of a work-flow for high-performance thin-layer chromatography data processing for untargeted metabolomics. J Planar Chromatogr Mod TLC.

[15] Ristivojević PM, Morlock GE (2016). The influence of preprocessing methods on multivariate image analysis in high-performance thin-layer chromatography fingerprinting. J Planar Chromatogr Mod TLC.

[16] Bessin A, Morlock G. Streamlined analysis of sugars in chicory root juice: comparison of two chromatographic methods. In: Morlock G, editor. HPTLC 2014, International symposium for high-performance thin layer chromatography, July 2–4, Lyon, France, Book of abstract. Giessen: Justus Liebig University; 2014. p. 51. http://www.hptlc.com/old/pdf/2014lyon/HPTLC_2014_final.pdf (abstract); http://www.hptlc.com/old/pdf/2014lyon/O28.pdf (slides). Accessed 7 Sep 2017.

[17] Abdel-Aal ESM, Hucl P (1999). A rapid method for quantifying total anthocyanins in blue aleurone and purple pericarp wheats. Cereal Chem.

[18] Oberlerchner JT, Fuchs C, Grausgruber H, Potthast A, Böhmdorfer S (2018). À côté calibration—making optimal use of time and space in quantitative high performance thin layer chromatography. J Chromatogr A.

[19] Friendly M (1991). SAS^®^ system for statistical graphics.

[20] Abdel-Aal ESM, Hucl P (2003). Composition and stability of anthocyanins in blue-grained wheat. J Agric Food Chem.

[21] Abdel-Aal ESM, Abou-Arab AA, Gamel TH, Hucl P, Young JC, Rabalski I (2008). Fractionation of blue wheat anthocyanin compounds and their contribution to antioxidant properties. J Agric Food Chem.

[22] Abdel-Aal ESM, Hucl P, Shipp J, Rabalski I (2016). Compositional differences in anthocyanins from blue- and purple-grained spring wheat grown in four environments in Central Saskatchewan. Cereal Chem.

[23] Zanoletti M, Abbasi Parizad P, Lavelli V, Cecchini C, Menesatti P, Marti A, Pagani MA (2017). Debranning of purple wheat: recovery of anthocyanin-rich fractions and their use in pasta production. LWT-Food Sci Technol.

[24] Li W, Pickard MD, Beta T (2007). Effect of thermal processing on antioxidant properties of purple wheat bran. Food Chem.

[25] Burešová V, Kopecký D, Bartoš J, Martinek P, Watanabe N, Vyhnánek T, Doležel J (2015). Variation in genome composition of blue-aleurone wheat. Theor Appl Genet.

[26] Gordeeva EI, Shoeva OY, Khlestkina EK (2015). Marker-assisted development of bread wheat near-isogenic lines carrying various combinations of purple pericarp (*Pp*) alleles. Euphytica.

[27] Bustos DV, Riegel R, Calderini DF (2012). Anthocyanin content of grains in purple wheat is affected by grain position, assimilate availability and agronomic management. J Cereal Sci.

[28] Ficco DBM, De Simone V, Colecchia SA, Pecorella I, Platani C, Nigro F, Finocchiaro F, Papa R, De Vita P (2014). Genetic variability in anthocyanin composition and nutritional properties of blue, purple, and red bread (*Triticum aestivum* L.) and durum (*Triticum turgidum* L. ssp. *turgidum* convar. *durum*) wheats. J Agric Food Chem.

[29] Liu MS, Wang F, Dong YX, Zhang XS (2005). Expression analysis of dihydroflavanol 4-reductase genes involved in anthocyanin biosynthesis in purple grains of wheat. J Integr Plant Biol.

[30] Knievel DC, Abdel-Aal ESM, Rabalski I, Nakamura T, Hucl P (2009). Grain color development and the inheritance of high anthocyanin blue aleurone and purple pericarp in spring wheat (*Triticum aestivum* L.). J Cereal Sci.

[31] Žofajová A, Pšenáková I, Havrlentová M, Piliarová M (2012). Accumulation of total anthocyanins in wheat grain. Agriculture (Pol’nohospodárstvo).

[32] Jing P, Noriega V, Schwartz SJ, Giusti MM (2007). Effects of growing conditions on purple corncob (*Zea mays* L.) anthocyanins. J Agric Food Chem.

[33] Tierno R, Ruiz de Galarreta JI (2016). Heritability of target bioactive compounds and hydrophilic antioxidant capacity in purple- and red-fleshed tetraploid potatoes. Crop Past Sci.

[34] Azuma A, Yakushiji H, Koshita Y, Kobayashi S (2012). Flavonoid biosynthesis-related genes in grape skin are differentially regulated by temperature and light conditions. Planta.

[35] Karppinen K, Zoratti L, Nguyenquynh N, Häggman H, Jaakola L (2016). On the developmental and environmental regulation of secondary metabolism in *Vaccinium* spp. berries. Front Plant Sci.

[36] Garg M, Chawla M, Chunduri V, Kumar R, Sharma S, Sharma NK, Kaur N, Kumar A, Mundey JK, Saini MK, Singh SP (2016). Transfer of grain colors to elite wheat cultivars and their characterization. J Cereal Sci.

[37] Varga M, Bánhidy J, Czeuz L, Matuz J (2013). The anthocyanin content of blue and purple coloured wheat cultivars and their hybrid generations. Cereal Res Commun.

[38] Rouau X, Mateo-Anson N, Barron C, Chaurand M, Lullien-Pellerin V, Mabille F, Samson MF, Abecassis J, Hemery Y (2010). Effet des procédés de fractionnement sur la composition et quelques propriétés nutritionnelles des produits céréaliers. Cah nutr diét.

[39] Aprodu I, Banu I (2012). Antioxidant properties of wheat mill streams. J Cereal Sci.

[40] Hemery Y, Rouau X, Lullien-Pellerin V, Barron C, Abecassis J (2007). Dry processes to develop wheat fractions and products with enhanced nutritional quality. J Cereal Sci.

[41] Brouns F, Hemery Y, Price R, Mateo Anson N (2012). Wheat aleurone: separation, composition, health aspects, and potential food use. Crit Rev Food Sci Nutr.

[42] Delcour JA, Rouau X, Courtin CM, Poutanen K, Ranieri R (2012). Technologies for enhanced exploitation of the health-promoting potential of cereals. Trend Food Sci Technol.

[43] Łata E, Fulczyk A, Kowalska T, Sajewicz M (2017). Thin-layer chromatographic method of screening the anthocyanes containing alimentary products and precautions taken at the method development step. J Chromatogr A.

[44] Reich E, Schibli A (2007). High-performance thin-layer chromatography for the analysis of medicinal plants.

[45] EDQM (European Directorate for the Quality of Medicines and Health Care). European Pharmacopoeia (Ph. Eur.), 9th ed., Chapter 2.8.25. High performance thin-layer chromatography. Strasbourg: Council of Europe; 2017. p. 295-8.

[46] Ambühl R. Pattern recognition in HPTLC fingerprints of medicinal plants. Master thesis, University of Basel. 2011. http://www.camag.com/media/5CCOS70X/Masterarbeit_RA2011_secured.pdf. Accessed 7 Sep 2017.

[47] Sherma J, Fried B (2003). Handbook of thin-layer chromatography.

[48] Wang J, Cao X, Qi Y, Ferchaud V, Chin KL, Tang F (2015). High-performance thin-layer chromatographic method for screening antioxidant compounds and discrimination of *Hibiscus sabdariffa* L. by principal component analysis. J Planar Chromatogr Mod TLC.

[49] Fichou D, Ristivojević P, Morlock GE (2016). Proof-of-principle of rTLC, an open-source software developed for image evaluation and multivariate analysis of planar chromatograms. Anal Chem.

[50] Böhmdorfer S, Genta-Jouve G, Grausgruber H, Rosenau T. HPTLC and clustering analysis for the classification of colored wheat varieties by anthocyanin patterns. Abstract ANYL-196, 249th ACS Annual Spring Meeting and Exhibition of the American Chemical Society, Denver, 2015. https://acswebcontent.acs.org/denver2015program/. Accessed 7 Sep 2017.

[51] Spangenberg B, Poole CF, Weins C (2011). Quantitative thin-layer chromatography.

